# Contraception and conception in Mid-life: a review of the current literature

**DOI:** 10.1186/s40695-017-0022-x

**Published:** 2017-08-16

**Authors:** Kristin Van Heertum, James Liu

**Affiliations:** University Hospitals MacDonald Women’s Hospital, 11100 Euclid Avenue, Cleveland, OH 44106 USA

**Keywords:** Mid-life, Mid-life contraception, Mid-life fertility, Perimenopause, Menopausal transition

## Abstract

In the United States, there are an increasing number of couples who are intentionally delaying child-bearing. As the average age of mothers continues to rise, more and more women are being faced with the difficulties of attempting conception at the various stages leading up to the menopausal transition. Not only do the chances of conception drastically decrease after the age of 40 years, but the probability of fetal loss (both early and late in pregnancy) significantly increases during this period as well. The aims of this review include providing an overview of the natural progression of the menopausal transition, examining the importance of appropriate contraception, and identifying the difficulties that women face when attempting conception during this physiologically dynamic stage of life. Finally, we will discuss the non-contraceptive benefits of contraception in preparation for pregnancy during the mid-life.

## Background

**Fig. 1:**
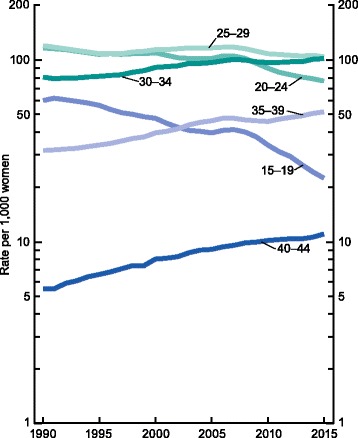
Birth rates, by age of mother: United States, 1990–2015. Reproduced from [3] (public domain). NOTE: Rates are plotted on a logarithmic scale. SOURCE: NCHS, National Vital Statistics System

Over the past several decades, the mean age of mothers in the United States has continued to increase [1, 2]. The mean maternal age at first birth in 1970 was 21.4 years, which increased to 24.9 years in 2000 and 26.3 years in 2014. This pattern was documented to occur across all birth orders. The birth rate for women age 40–44 years has continued to increase over the last 3 decades (Fig. 1). For example, in 2015, the birth rate in this age group was 11 births per 1,000 women, up 4% from the year prior [3]. More strikingly, the number of births in the U.S. occurring in women who are ≥ 45 years rose from 0.3 births per 1,000 women in the early 1990’s to 0.8 births per 1,000 women in 2013 [4, 5]. As women continue to further delay childbearing, more couples may be affected by the natural fertility decline that occurs in the mid-life. In this review, we will discuss the physiological decline in ovarian reserve and the resulting decrease in fecundity and fecundability, as well as the need for contraception during this phase; the risks of pregnancy with advancing maternal age; and the options for fertility treatment and family planning in this age group.

## Physiology of menopause

Menopause is defined as the absence of menses for one year due to the depletion of ovarian reserve [6]. The median age of natural menopause in the United States is 51.4 years [7]. Prior to the final menstrual period, there are a variety of hormonal changes that occur in relation to the dwindling number of functional ovarian follicles. The earliest hormonal change in the menopausal transition is the gradual decrease in serum inhibin B levels [8, 9]. This is followed by a gradual increase in serum FSH levels and ultimately a subsequent decrease in circulating estradiol levels.

The Daily Hormone Study (DHS), a part of the Study of Women’s Health Across the Nation (SWAN), examined daily urine collections for up to 50 days annually for 3 years in 848 women aged 43–53 years [10]. The aim of this study was to document declining luteal function through the menopausal transition by measuring urinary LH, FSH, and metabolites of both estrogen and progesterone. There was a significant decrease in the number of ovulatory cycles documented over the 3-year study period (80.9% → 64.7%); additionally, there was an increased number of anovulatory cycles with no bleeding, which was associated with progression to early perimenopause (OR = 2.66) or late perimenopause (OR = 56.21). The number of anovulatory cycles that were associated with a bleeding episode did not change over the course of the study (approximately 10%) and were not associated with progression to perimenopause. However, pregnanediol glucuronide, a surrogate measure for serum progesterone levels, decreased by 6.6% each year. Thus, the study documented a decrease in luteal progesterone, albeit small, even in ovulatory cycles as well as a decrease in the proportion of ovulatory cycles across the progression through the menopausal transition. In addition, the study demonstrated that anovulatory cycles without a bleed were relatively hypoestrogenic compared with anovulatory cycles associated with a bleeding episode. The study also found that both low and high early follicular-phase estradiol were predictive of progression in the menopausal transition. The proposed decrease in ovulatory cycle progesterone production may contribute to the decreased fecundability that has been well-documented in this age group. It has been hypothesized that this progressive deficiency in the luteal phase is a result of impaired folliculogenesis [11]. As inhibin B decreases, FSH not only increases overall, but starts to increase earlier in the luteal phase of the preceding cycle [12–14]. This leads to earlier follicular recruitment, a shortened follicular phase and abnormal folliculogenesis, that leads to a defective luteal phase.

These functional shifts in the pattern of folliculogenesis result in alterations of the length of menstrual cycles. As women age, they may experience either shortening or lengthening of their cycles due to a shortened follicular phase, impaired folliculogenesis, and an increasing number of anovulatory cycles. The clinical correlates to these physiologic alterations are the hallmark symptoms of menopause: hot flushes (hypothalamic response to decreased estrogen levels), sleep disturbances (often associated with hot flushes), and depressed mood [6, 15].

## Fecundability in the Mid-life

**Fig. 2:**
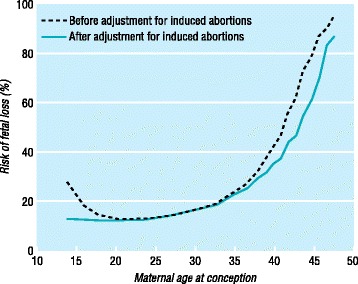
Risk of fetal loss by maternal age at conception. Reproduced with permission from [17]

**Fig. 3:**
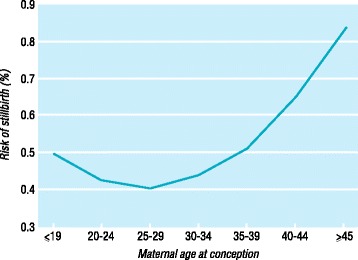
Risk of stillbirth by maternal age. Reproduced with permission from [17]

Evaluation of fecundity, defined as the fertility potential, can be assessed by highly sensitive urine hCG testing. In a study of married women not using contraception in rural Bangladesh tested for the presence of urinary hCG twice weekly, the probability of conceiving in a single menstrual cycle began to significantly decline in the early 40’s [16]. However, the fecundity rate, the probability of achieving live birth in a single menstrual cycle, is further decreased by an increasing rate of fetal loss with increasing maternal age. A study from the Danish Health Service was able further stratify the increasing risk of fetal loss with maternal age. Anderson et al. 2000 queried fetal loss data from the civil registration system in Denmark from 1978 to 1992 [17]. The study documented a miscarriage risk of 8.9% at ages 20–24 years, 54.5% at age 42 years, and 74.7% in women aged 45 years or older (Fig. 2). The risk of fetal loss significantly increased after age 35 years, with a risk of greater than 20% at age 35 years. The incidence of stillbirth was also found to rise after age 35 years (Fig. 3).

## Prevention of unintended pregnancy

Despite the decreasing fecundity that comes with increasing age, women in the midlife still need reliable contraception. Currently, the American College of Obstetricians and Gynecologists recommends continuation of contraception until 50–55 years of age in those women who wish to prevent pregnancy [18]. In 2006, the overall rate of unintended pregnancy in the United States was 49%, with 48% of those being in women age 40–44 years [19]. It is difficult to estimate how many women in this age group are at risk for unintended pregnancy. However, one study from Europe examining data from five different countries estimated that approximately 30% of women ages 45–49 years were not using any form of contraception [20]. Another study, which used data from the Massachusetts Behavioral Risk Factor Surveillance System, found that 14.7% of women ages 40–44 years and 16.8% of women ages 45–50 years who were at risk for unintended pregnancy were not using any form of contraception [21].

Unintended pregnancy is of particular concern when women are suffering from comorbid conditions that would not only result in significant health risk if they were to become pregnant, but could even result in death. Such conditions include hypertension, diabetes and heart disease, which have an increasing prevalence with advancing age [22]. While the overall maternal mortality rate in the U.S. has continued to increase over the years, the most substantial increase has been noted in women age 45 and older. In 1993 there were no reported deaths from 2,329 live births in the U.S. in this age group, while in 2014 there were 171 maternal deaths reported in 8,443 live births [23].

There is also a higher risk of pregnancy-specific complications with advancing age. Women over the age of 44 have a greater risk of gestational diabetes, cesarean delivery, pregnancy-related hypertensive disorders and fetal aneuploidy [24]. Generally, any form of contraception is felt to be safe for women age 45 years and older, provided that they do not have other risk factors [22]. However, as women age, the incidence of comorbidities such as hyperlipidemia, hypertension, heart disease, stroke, venous thromboembolism, and diabetes, increases. These conditions can preclude the use of estrogen-containing forms of contraception, however other forms of contraception are considered safe alternatives in these patients, including intrauterine devices (IUDs) and progestin-only implants.

## Noncontraceptive benefits of contraceptives

Hormonal contraceptives offer a variety of benefits beyond pregnancy prevention. Any form of hormonal contraception, including combined oral contraceptives (COCs) and progestin-only contraceptives (progestin-only pill, injection, implant or IUD) decreases the amount of menstrual blood loss experienced by women and can be used as a temporizing agent in women suffering from heavy menses [25]. This can be a useful approach in patients who wish to avoid surgery, particularly to maintain their fertility potential. Additional benefits of hormonal contraceptives include reductions in both endometrial and ovarian cancer risk. Both COCs and depot medroxyprogesterone acetate have been found to significantly reduce the risk of endometrial cancer. This effect may last for up to 20 years following cessation of treatment [26–29]. The levonorgestrel intrauterine system can provide local progestin action with minimal systemic effects. This type of IUD is an effective treatment for endometrial hyperplasia without atypia, and will decrease endometrial cancer risk [30, 31]. A meta-analysis of data from 45 epidemiologic studies of women with ovarian cancer versus controls showed a reduction in ovarian cancer risk of 27% with the use of COCs [32].

Another condition that is frequently treated with hormonal contraceptives is endometriosis. The reported incidence of endometriosis varies depending on the study population; however, approximately 10% of reproductive age women will have endometriosis [33]. This incidence is higher in those presenting with pain or infertility. Conversely, 30-50% of women with endometriosis with have sub- or infertility [34]. The efficacy of in vitro fertilization (IVF) appears to be decreased in patients with endometriosis. A 2002 meta-analysis of studies comparing IVF outcomes in women with endometriosis versus tubal factor showed a significantly lower pregnancy rate in patients with endometriosis (odds ratio 0.56; 95% confidence interval 0.44-0.7), with even lower pregnancy rates in women with severe endometriosis compared to those with mild disease (odds ratio 0.6; 95% confidence interval 0.42-0.87) [35]. Women with endometriosis are often treated with COCs or progestin-only therapy to suppress their disease and provide pain relief. These medical suppressive therapies, however, prevent ovulation and conception.

A Cochrane review examined randomized trials comparing GnRH agonist, danazol and COCs with placebo in patients with a diagnosis of endometriosis. The study found no difference in spontaneous pregnancy rates with any treatment versus placebo [36]. The American Society of Reproductive Medicine (ASRM) does not recommend delaying infertility treatment with medical suppression of endometriosis as there is no improvement in pregnancy rates [37]. On the other hand, another Cochrane review showed that pre-treatment with prolonged GnRH agonist therapy may improve IVF outcomes [38].

## Planning for conception in mid-life

Women who wish to discontinue contraception and attempt to conceive may experience a variable length of time to resumption of normal menses. Barnhart et al. examined the time to pregnancy after discontinuation of a continuous regimen of levonorgestrel 90 μg and ethinyl estradiol 20 μg [39]. The study found pregnancy rates of 57%, 81%, and 86% at 3, 12 and 13 months, respectively, suggesting there is no significant delay in the return to fertility with continuous OC regimens. However, these women were all 35 years or younger. Therefore, this information may not be generalizable to the population in question in this review.

## Third-party reproduction

The likelihood of a successful live birth in older women, particularly over the age of 45, is low. Even with IVF, the oocyte yield and quality will be poor in patients over the age of 42 [40]. Donor oocyte remains a reliable way to significantly increase the chance of live birth in women at advancing ages, though even this does not provide a 100% chance of success. Current studies show that with donor oocytes, older women have success rates similar to the age of the oocyte donor [41]. Alternatively, many women may be without a male partner for various reasons. Donor intrauterine inseminations could be a viable option in these patients.

## Conclusions

The negative effect of age on reproductive potential is well known. As women approach menopause, it is important that they continue to use reliable contraception to reduce the risk of unintended pregnancy. Once patients are ready to conceive, they need to receive adequate pre-conception counseling regarding their risks in pregnancy, risk of fetal loss, and raising a child at an older age. Women of advancing age often suffer from infertility, so there should be no delay in referring them to a fertility specialist. These women may require more aggressive therapies such as superovulation, IVF or donor oocyte and/or sperm.

## Abbreviations

ASRM: American Society of Reproductive Medicine
COC: Combined oral contraceptives
DHS: Daily hormone study
FSH: Follicle-stimulating hormone
IUD: Intrauterine device
IVF: In vitro fertilization
LH: Luteinizing hormone
SWAN: Study of Women’s Health Across the Nation
